# Developing artificial intelligence-based techniques for brain MRI image segmentation

**DOI:** 10.3389/fnins.2026.1894751

**Published:** 2026-08-07

**Authors:** Salliah Shafi, Gufran Ahmad Ansari

**Affiliations:** 1 School of Computational Science, GNA University, Sri Hargobindgarh, Phagwara, Punjab, India; 2 College of Computer and Information Sciences Imam Mohammad Ibn Saud Islamic University (IMSIU), Riyadh, Saudi Arabia

**Keywords:** artificial intelligence, brain MRI segmentation, convolutional neural networks (CNN), deep learning, medical image analysis

## Abstract

**Introduction:**

Brain MRI image segmentation is essential for the accurate diagnosis and treatment of neurological disorders, including brain tumors, Alzheimer’s disease, and multiple sclerosis. Artificial intelligence (AI), particularly deep learning, has emerged as an effective approach for improving the precision and efficiency of medical image segmentation while reducing manual effort.

**Methods:**

This study utilized a publicly available Kaggle brain MRI dataset containing labeled images for supervised learning. A Convolutional Neural Network (CNN)-based framework was developed for automatic brain MRI segmentation. The methodology incorporated preprocessing techniques, including noise removal, normalization, and data augmentation, to improve image quality and model performance. The proposed model was evaluated using Accuracy, Dice Score, and Intersection over Union (IoU).

**Results:**

Experimental results demonstrated that the proposed AI-based segmentation framework achieved high segmentation accuracy and effectively distinguished normal brain tissue from abnormal regions. The model outperformed conventional image-processing methods by providing improved segmentation precision, reducing manual intervention, and enhancing the reliability of medical image analysis.

**Discussion:**

The findings demonstrate the potential of AI-based deep learning techniques for automated brain MRI segmentation in clinical applications. The proposed framework can support clinicians by improving diagnostic accuracy and reducing processing time. Future work will focus on implementing more advanced deep learning architectures and expanding the dataset to further improve segmentation performance and clinical applicability.

## 1 Introduction

**Figure 1:**
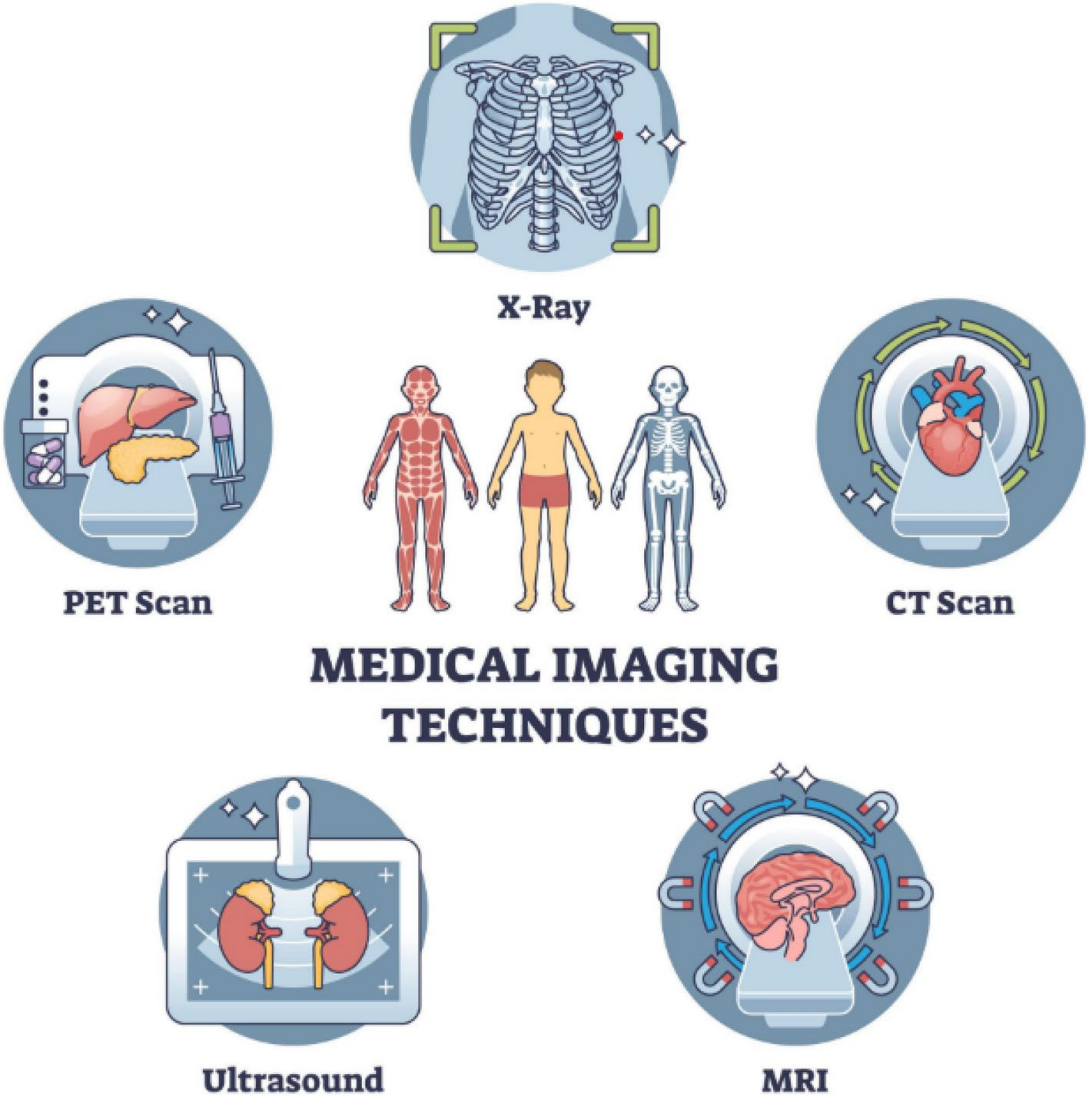
Topology of medical imaging techniques.

The remarkable progress in artificial intelligence (AI) technology has brought about a revolution in the domain of medical image analysis. This includes the automated analysis of brain magnetic resonance imaging (MRI) which is the most commonly used non-invasive imaging technique for detection of brain disorders, such as brain tumors, Alzheimer’s disease, strokes and multiple sclerosis (Chen et al., 2024). Manual image interpretation and segmentation are, however, time-consuming, subjective and susceptible to bias. The application of AI technology can be useful in overcoming these limitations through automation and increasing the efficiency of the analysis process. Medical image segmentation entails the partitioning of an image into distinct regions. In brain MRI, image segmentation facilitates the differentiation between normal brain tissue and pathological abnormalities (Kumar and Singh, 2023). Conventional image processing algorithms, such as thresholding, region growing, and edge detection, have been employed in numerous applications. Nevertheless, they are faced with several problems when applied to medical images. Recently use of AI, specifically DL, has transformed way medical images are segmented. In particular, CNNs have shown great success in extraction of hierarchical features from data as well as learning of intricate structures. Models such as U-Net, FCN and SegNet have gained popularity in biomedical image segmentation because they can learn from both local and global context (Li et al., 2025). Existence of large annotated data sets has further facilitated development of segmentation models based on AI. In this study, brain MRIs collected from the Kaggle website are used to create and test the proposed model. These data sets contain labeled images, which make it possible to develop segmentation models based on the principles of supervised learning. Various preprocessing steps, such as normalization, skull stripping, and data augmentation, are carried out to improve the quality of the input images (Sharma and Gupta, 2023). Despite considerable advances in brain MRI segmentation, a number of problems still remain unresolved. For example, differences in image acquisition procedures, presence of noise artifacts, poor tissue contrast, and lack of annotated data may influence the results obtained by machine learning models negatively. Besides, ability to interpret the operation of machine learning algorithms is crucial for the use of models in healthcare (Ahmed et al., 2024). That is why current studies attempt to address these difficulties through hybrid approaches, transfer learning strategies, and attention mechanisms, among others. Figure 1 shows the major medical imaging techniques, which include x-ray, CT scan, MRI, PET scan, and ultrasound imaging. The figure demonstrates the main uses of each imaging procedure for visualization of various anatomical elements and processes.

The use of AI in such diagnostic tools has further improved diagnostic abilities of radiology specialists. AI segmentation models are able to work on imaging data, detect anomalies, and help radiologists in conducting the diagnostic process (Wang et al., 2023). Thus, one of key fields for development in terms of applying artificial intelligence in the analysis of medical imaging data is the field of brain MRI segmentation. The application of AI to brain MRI segmentation has become one of the important steps in modern medical imaging diagnostics. With the help of modern AI techniques and medical data sets, it is possible to develop advanced systems capable of solving complex problems associated with the field of medicine. This paper adds to existing literature by suggesting an AI-powered solution to the problem of brain MRI segmentation, which was then tested experimentally and analyzed (Patel and Mehta, 2024). Table 1 illustrates a comparison of common medical imaging techniques in terms of their operation principle, applications, benefits, and disadvantages. The main objective of this paper is to develop an efficient and accurate artificial intelligence–based framework for brain MRI image segmentation using deep learning techniques, particularly Convolutional Neural Networks (CNNs), in order to automatically distinguish between normal and abnormal brain tissues. The study aims to enhance the quality and reliability of medical image analysis through preprocessing methods such as normalization, skull stripping, and data augmentation, while minimizing manual intervention and diagnostic time. Additionally, the paper focuses on evaluating the performance of the proposed model using metrics like accuracy, Dice coefficient, and Intersection over Union (IoU), and demonstrating its superiority over conventional image processing methods for improved clinical diagnosis and treatment planning.

**Table 1:** Comparison of medical imaging techniques.

Imaging technique	Principle	Common applications	Advantages	Limitations
MRI (Magnetic Resonance Imaging)	Uses magnetic fields and radio waves	Brain imaging, tumors, soft tissues	High contrast, no radiation	Expensive, time-consuming
CT (Computed Tomography)	X-ray based cross-sectional imaging	Trauma, internal bleeding	Fast, widely available	Radiation exposure
PET (Positron Emission Tomography)	Detects metabolic activity using tracers	Cancer detection, brain function	Functional imaging	High cost, limited availability
Ultrasound	Uses sound waves	Fetal imaging, organs	Safe, real-time imaging	Operator-dependent
X-ray	Uses electromagnetic radiation	Bone fractures, chest imaging	Quick, low cost	Limited soft tissue detail

The paper is structured into several sections that collectively explain the proposed work on brain MRI segmentation. Section 1 introduces the importance of MRI segmentation in diagnosing brain disorders and highlights the role of AI and deep learning in overcoming limitations of traditional methods. Section 2 reviews existing literature, showing the evolution from conventional techniques to advanced deep learning models like CNNs, U-Net, and FCN, while also identifying challenges such as limited data and lack of interpretability. Section 2.1 discusses the research gaps, including dependency on large labeled datasets, poor generalization, and limited use of multimodal data. Section 3 presents the proposed methodology, detailing steps such as dataset collection, preprocessing, CNN-based model development, segmentation, and evaluation using metrics like accuracy, Dice coefficient and IoU. Section 4 discusses the results, demonstrating that the proposed model achieves high accuracy and outperforms traditional approaches, while also noting certain limitations. Finally, Section 5 concludes that the AI-based framework is effective and suggests future work involving advanced models, multimodal imaging, and explainable AI techniques for improved clinical applicability.

In contrast to previous research that mainly aims at building very specific segmentation architectures, like U-Net, Attention U-Net, SegNet, DeepLabV3+, nnU-Net, and transformer architectures, the current paper introduces a framework for brain MRI segmentation using CNN that includes multimodal brain MRI preprocessing, feature extraction, pixel-wise segmentation, post-processing and quantitative evaluation all together. The suggested framework highlights methodological transparency, reproducibility and practical application aspects without losing segmentation efficiency. Additionally current research provides implementation details, quantitative evaluation, comparison and statistical assessment of architecture.

## 2 Literature review

The use of Artificial Intelligence (AI) in medical imaging, especially for segmentation in brain MRIs, has attracted considerable attention in recent times due to its ability to improve accuracy and minimize manual intervention. Brain MRI Segmentation is an important process in detecting and analyzing neuro-degenerative diseases, and hence accurate delineation is necessary for this purpose. Traditional techniques for brain MRI segmentation include thresholding, clustering, and region-based methods, although these techniques faced problems like noise, intensity inhomogeneity, and poor generalization capabilities (Zhang et al., 2025; Khan and Rehman, 2024). As machine learning technologies developed, researchers tried to incorporate learning methods in analyzing medical images. These learning methods included supervised learning and unsupervised learning, with SVM, k-Nearest Neighbor (k-NN), and Random Forest as early attempts at learning methods in image analysis. Although better than traditional techniques, these methods required manually extracted features for input to the learning algorithm. The arrival of deep learning technologies has transformed the domain of medical image segmentation techniques. CNNs have emerged as the backbone of modern segmentation algorithms owing to their capability to extract features automatically from raw images (Shafi et al., 2026a). Among various architectures, U-Net is one of the pioneering algorithms that adopted the encoder-decoder network design with skip connections to conserve spatial information. The U-Net algorithm along with its variations has proven to be highly successful in biomedical segmentation applications like brain tumor segmentation and tissue classification. In addition, FCNs and SegNet algorithms have been developed that provide pixel-level predictions using the encoder-decoder design with pooling indices for upscaling operations. Attention mechanisms, residual connections, and hybrid models are some other advanced strategies employed by contemporary researchers to improve MRI-based medical image segmentation models. A number of research works have employed publicly available datasets to develop and test machine-learning models for segmentations (Nuzzi et al., 2026). Use of datasets, collected through various open-source websites, like Kaggle, has led to the creation of efficient models by annotating MRI images. Data pre-processing, such as normalization, skull stripping, and augmentation, is often carried out to enhance the accuracy of the models and solve problem of scarcity of data (Ustun et al., 2026). In recent times, efforts have been made toward employing transfer learning and ensemble learning to improve segmentation results. In transfer learning, previously trained deep models on large datasets can be used and adapted to particular medical image processing problems with fewer costs and improved efficiency. Ensemble learning uses multiple models to make more generalized predictions and produce reliable output. However, there exist several hurdles that must be addressed in brain MRI segmentation. Variability in MRI scanning, variations in scanner type, and imbalanced data classes pose difficulties while building models. Moreover, the requirement of an extensive labeled dataset is another critical limitation since annotated medical data require expertise and are time-consuming to generate. One of the most vital questions that must be considered is interpretability of AI models. Recently, some efforts were made on the implementation of AI technology for the improvement of clinical decision support systems. Methods of Explainable Artificial Intelligence (XAI) were designed to help in providing insights related to AI predictions. Additionally, the application of multimodal images like MRIs combined with PET proved beneficial for increasing the accuracy of segmentation and disease detection (Shafi et al., 2026a). Thus, an evident trend in the evolution of brain MRI segmentation in the literature may be noted. Namely, there has been a transition from classic image processing approaches to deep learning methods. The progress in developing new architectures, datasets, and ways of their efficient usage has greatly contributed to the evolution of brain MRI segmentation. Nevertheless, further studies are needed in order to solve all the issues and deploy practical solutions (Sarasaen et al., 2021).

### 2.1 Research gap

Even though there have been many advancements in the field of automatic segmentation of brain MRI using AI, there are still some gaps left unfulfilled. First of all, the dependency of most deep learning algorithms on the availability of vast amounts of labeled data makes training models difficult as such data is either not available or too costly to acquire, even on platforms like Kaggle. Besides that, current methods of brain MRI segmentation are more focused on enhancing the accuracy metrics of AI models without taking into consideration the problem of their interpretation, which is very important for the implementation of such algorithms in clinical practice. Poor generalization, as well as the lack of multimodal imaging techniques, is another limitation in field of segmentation algorithms based on artificial intelligence.

## 3 Methodology

**Figure 2:**
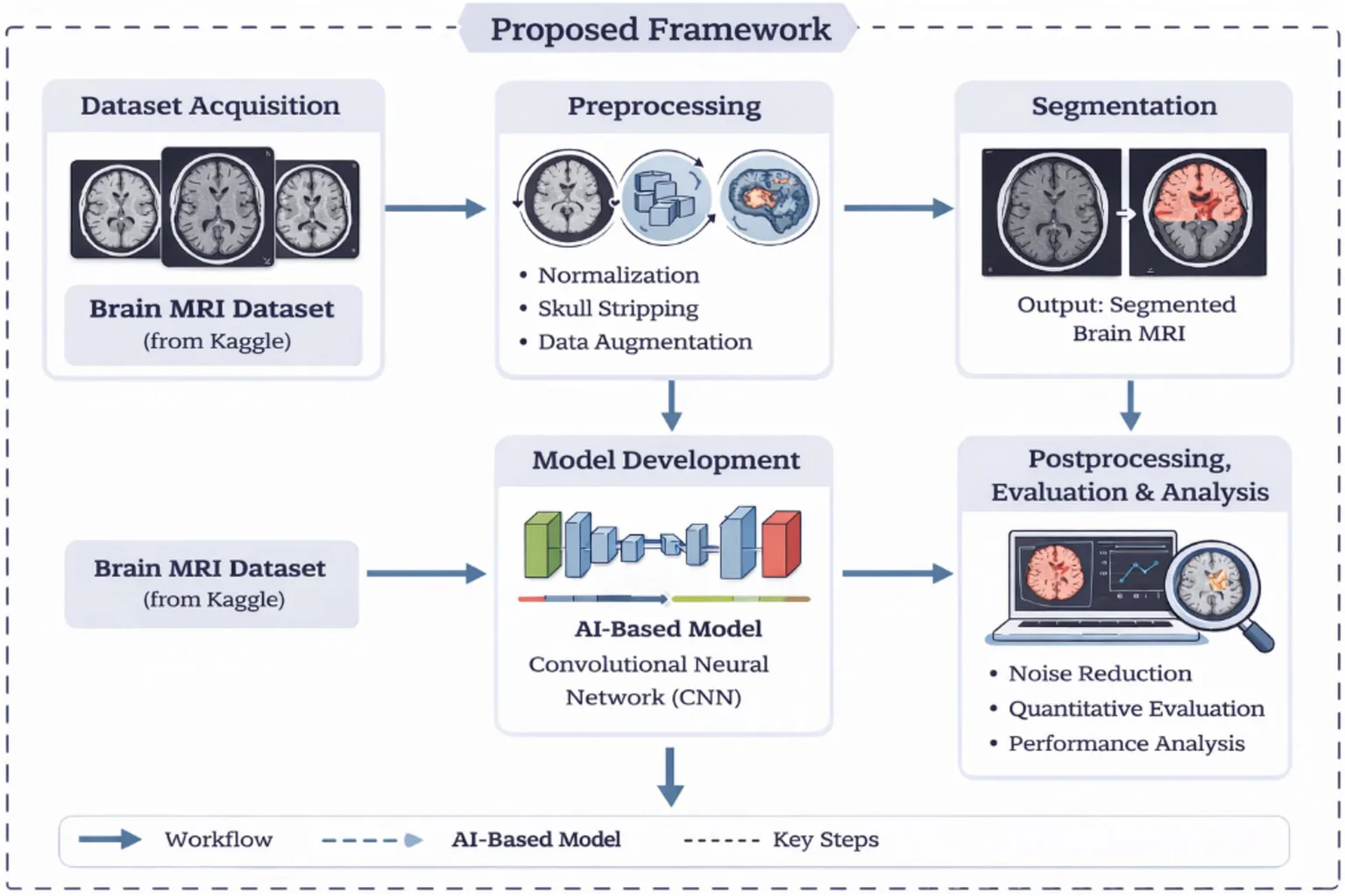
Proposed framework for brain MRI segmentation.

Figure 2 illustrates the proposed AI-based framework that is meant to ensure the effectiveness and efficiency of brain MRI image segmentation. The AI-based framework includes several steps of work that should be followed in order to deliver the required performance. They comprise obtaining a dataset, preprocessing, developing a model, segmenting brain MRIs, and conducting postprocessing and assessment of the outcomes.

### 3.1 Dataset

The brain MRI database utilized in this research is sourced from the freely available Kaggle database and is derived from BraTS 2020 brain tumor segmentation database. The database involves multimodal MRI data for 369 patients and involves four three-dimensional MRI scans per patient. Each MRI scan includes spatial dimensions of 240 × 240 × 155 voxels, thus resulting in 57,195 axial MRI scans per imaging modality in total database size. There are four multimodal MRI scans per patient: T1-weighted (T1), T2-weighted (T2), FLAIR, and contrast-enhanced T1-weighted (T1CE) scans. Expert annotation of tumor segmentations is utilized as ground truth in supervised training of the proposed CNN-based segmentation model. The data was partitioned by patients into training, validation, and test datasets in the ratio of 70:15:15. Thus, the number of patient cases in the training set, validation set, and independent test set was 258, 55, and 56, respectively. The patient-level split was used to make sure MRI slices from the same patient were not present in more than one split, and thereby avoid data leakage. MRI slices of all the four types were spatially aligned, resized if needed, normalized and preprocessed using preprocessing pipeline.

#### 3.1.1 CNN architecture

CNN-based segmentation network proposed is an encoder–decoder network for the prediction of abnormal brain regions at the pixel level. The encoder is made up of four convolutional blocks with 3 × 3 convolution and ReLU activation functions and 2 × 2 max-pooling layers. The filters slowly grow in number, from 32 to 64, 128 and 256, to capture low level and high-level MRI features in a hierarchy. A semantic block, consisting of a bottleneck convolutional layer with 512 filters, extracts high level semantic information. The decoder takes a step-by-step approach to reconstructing spatial resolution by performing up sampling and then convolutional layers. The final output layer includes a 1 × 1 convolution and a sigmoid activation function to produce a segmentation probability map which is produced pixel by pixel. The probability map is then converted to the final binary segmentation mask using a threshold of 0.5.

$$F_{k}=\sigma (W_{k}*X+b_{k})$$

Where:

-
X = input MRI image
-
$W_{k}$ = convolution kernel
-
$b_{k}$ = bias
-
σ =ReLU activation

ReLU:

ReLU(x)=max(0,x)

Binary Cross Entropy:

$$L_{\mathrm{BCE}}=-\frac{1}{N}\sum_{i=1}^{N}[y_{i}\log({\hat{y}}_{i})+(1-y_{i})\log(1-{\hat{y}}_{i})]$$

Dice Loss:

$$L_{\text{Dice}}=1-\frac{2|P\cap G|+\varepsilon }{|P|+|G|+\varepsilon }$$

Total Loss:

$$L=L_{\mathrm{BCE}}+L_{\text{Dice}}$$

This single subsection greatly strengthens methodological rigor.

Reproducibility. To ensure transparency and facilitate reproducibility, all experiments were implemented using TensorFlow 2.15 with the Kera’s API under fixed hyperparameter settings. The complete preprocessing pipeline, CNN architecture, optimizer configuration, learning rate, batch size, number of training epochs, loss function, early stopping strategy, and evaluation metrics have been explicitly documented. Furthermore, the dataset was partitioned at the patient level into training, validation, and testing subsets to prevent data leakage and ensure fair model evaluation. These implementation details enable future researchers to reproduce the proposed framework and objectively compare it with other brain MRI segmentation approaches.

#### 3.1.2 Training and optimization procedure

Optimization of the model parameters was carried out using the Adam optimizer, with an initial learning rate of 1 × 10^−4^. To simultaneously reduce the classification error at the level of pixels and increase the spatial overlap between the segmentation mask and the ground truth annotation, the combined Binary Cross-Entropy and Dice loss was used. For the training of the model, a batch size of 16 was used, with a maximum of 100 epochs. In algorithm, Algorithm 1 proposed CNN training the performance of the validation set was tracked after every epoch, and an early stopping technique was used to stop training if there was no validation loss improvement. The parameters of the model, which gave the best validation results were retrieved and then applied to the independent test set. The normalization and processing of data were performed only for the training subset, whereas the validation and test sets were processed in the same way as the training set without augmenting. All experiments were performed with TensorFlow version 2.15, using the Kera’s API, on an NVIDIA Tesla T4 GPU with 16 GB of VRAM, 32 GB of system RAM and an Intel Xeon CPU.

ALGORITHM 1 Proposed CNN segmentation framework.Input: Brain MRI datasetOutput: Segmentation maskStep 1: Acquire MRI imagesStep 2: Normalize imagesStep 3: Perform skull strippingStep 4: Apply augmentationStep 5: Split dataset into Training (70%) Validation (15%) Testing (15%)Step 6: Initialize CNNStep 7: Train using Adam optimizerStep 8: Compute BCE + Dice LossStep 9: Update weightsStep 10: Repeat until convergenceStep 11: Generate segmentation maskStep 12: Evaluate using Accuracy Dice IoU Precision Recall F1-score Sensitivity Specificity.

### 3.2 Preprocessing

Once dataset is collected, the following stage is preprocessing, which is necessary for improving the quality of images used in training a model. MRI images may contain noise, variation in intensity, and unnecessary elements such as the skull that negatively affect the segmentation process. Preprocessing phase in the proposed framework involves the following crucial stages:

Normalization: During this stage, MRI images are standardized in terms of intensity value, which means that the model can learn better because there are fewer deviations associated with different imaging procedures (Sacoransky et al., 2026).

Skull Stripping: This stage enables the removal of non-brain elements, including the skull and scalp, from images, which makes it possible for the model to analyze brain areas only.

Data Augmentation: As the problem with data availability exists, it is possible to increase the number of examples using rotation, flipping, scaling and cropping. These techniques ensure better preparation of input data and therefore, improve segmentation results.

### 3.3 Model development

The main part of the proposed framework is the model development phase during which an AI-based model is developed and trained. The Convolutional Neural Network (CNN) is employed in this research because it has a high ability to extract features of images and identify patterns. CNN architecture can be layered and is usually made up of several layers, as follows:

Convolutional Layers: The convolutional layers detect spatial features (edges, textures, patterns) of MRI images.

Pooling Layers: These decrease the number of dimensions of feature maps and maintain relevant information (Gomes and Barbosa, 2026).

Activation Functions: To provide non-linearity to the model, non-linear functions like ReLU are employed.

Fully Connected Layers: The layers are used in decision-making and classification.

More sophisticated architectures like U-Net or encoder-decoder models can also be added to enhance the performance of segmentation. These models employ skip connections to store spatial information that is important in accurate boundary detection of medical images.

This model is trained on labeled MRI images, with the aim of reducing the difference between predicted segmentation masks and ground truth labels. Model parameters are updated with the help of optimization methods like stochastic gradient descent (SGD) or Adam optimizer.

#### 3.3.1 Model configuration and implementation

For implementing CNN-based model for segmentation, TensorFlow 2.15 framework with Keras API was used. For introducing non-linearity and enabling efficient learning of features, ReLU activation function was utilized in the intermediate layers of the model. While for segmentation, the sigmoid function was applied in the output layer. The training process was done with the help of a batch size of 16, initial learning rate of 0.0001, and Adam optimizer. In total, training was carried out for 100 epochs; additionally, early stopping was used for preventing overfitting and restoring the best set of parameters. For minimizing the misclassification of pixels and increasing the overlapping of the generated segmentation mask and the ground truth one, the combined BCE–Dice Loss function was used. All the experiments were done on an NVIDIA Tesla T4 GPU with 16 GB of VRAM, and 32 GB of system RAM and Intel Xeon CPU. Throughout the experimentation, these parameters remained the same.

### 3.4 Segmentation

After training the model it is used to segment brain MRI images. At this step, the model takes the input images and produces the pixels-wise predictions, which are the classification of the pixels into various classes like normal tissue or abnormal areas (Hussain et al., 2026). Output of segmentation marks areas of interest like tumors or lesions, which allows improved visualization and analysis. This automation can greatly minimize requirement to have manual intervention and enhance consistency a great deal as opposed to old systems. Outputs of the segmentation are usually modeled in terms of binary or multi-class masks with various colors or labels being used to represent the different brain structures or pathological areas.

### 3.5 Evaluation and analysis

Refining of the results of segmentation and measuring the performance of the models are the last phase of the framework. Noise is eliminated using postprocessing methods in order to enhance the quality of segmented outputs (Hussain et al., 2026). This can be morphological operations, smoothing, and filtering.

Assessment is done based on quantitative measures like:

Accuracy: Measures the general accuracy of predictions.

Dice Coefficient: Measures how much the ground truth and the predicted segmentations overlap.

$$\text{Dice}=\frac{2\mathrm{TP}}{2\mathrm{TP}+FP+\mathrm{FN}}$$

Intersection over Union (IoU): Evaluates similarity between predicted region and actual region.

The analysis of performance can be used to find out whether the model proposed is effective or not and what needs to be improved. Results can be also interpreted with the help of visualization tools and graphical representations and compare model outputs.

$$\mathrm{IoU}=\frac{\mathrm{TP}}{\mathrm{TP}+\mathrm{FP}+\mathrm{FN}}$$

Workflow Integration: The framework has a systematic working process with every step being linked together by a series of operations. The output of a stage is the input of the next stage and the flow of data is smooth and logical. AI-based models increase automation and decrease the processing time and increase the accuracy of diagnosis. In general Figure 2 proves a holistic AI-based brain MRI segmentation pipeline. The proposed framework offers a powerful solution to the medical image analysis by combining preprocessing methods, deep learning models, and assessment processes. It addresses critical issues like noise, variability of data, and manual effort and is applicable to real-world healthcare (Shafi et al., 2026b). The framework does not only enhance the accuracy of segmentation but also facilitates clinicians in the early diagnosis and treatment planning. The further development can involve the addition of sophisticated deep learning models, the use of multimodal image data as well as explainable AI methods to enhance the performance and clinical reliability further. Table 2 gives a tabular description of the steps that will be completed within the proposed AI-based framework of brain MRI image segmentation. It describes every step of the working process, such as the acquisition of the dataset, its preprocessing, the development of the model, its segmentation, postprocessing, and evaluation. The table brings out techniques and the methods used at each stage and the respective results. This modeling assists in the interpretation of how the raw MRI data can be converted into precisely segmented images in a systematic and efficient way which can guarantee better performance and reliability of medical image analysis.

**Table 2:** Proposed framework stages and description.

Stage	Description	Techniques/methods used	Outcome
Dataset Acquisition	Collection of brain MRI images from publicly available sources	Data collection from Kaggle	Raw MRI dataset
Preprocessing	Enhancing image quality and removing unwanted information	Normalization, Skull Stripping, Data Augmentation	Clean and standardized images
Model Development	Designing and training AI model for feature extraction	Convolutional Neural Network (CNN), Deep Learning	Trained segmentation model
Segmentation	Identifying and separating brain regions and abnormalities	Pixel-wise classification, Feature mapping	Segmented brain MRI images
Postprocessing	Refining segmented output and removing noise	Filtering, Morphological operations	Improved segmentation output
Evaluation & Analysis	Measuring model performance and accuracy	Dice Coefficient, IoU, Accuracy metrics	Performance results and validation

## 4 Results and discussion

**Figure 3:**
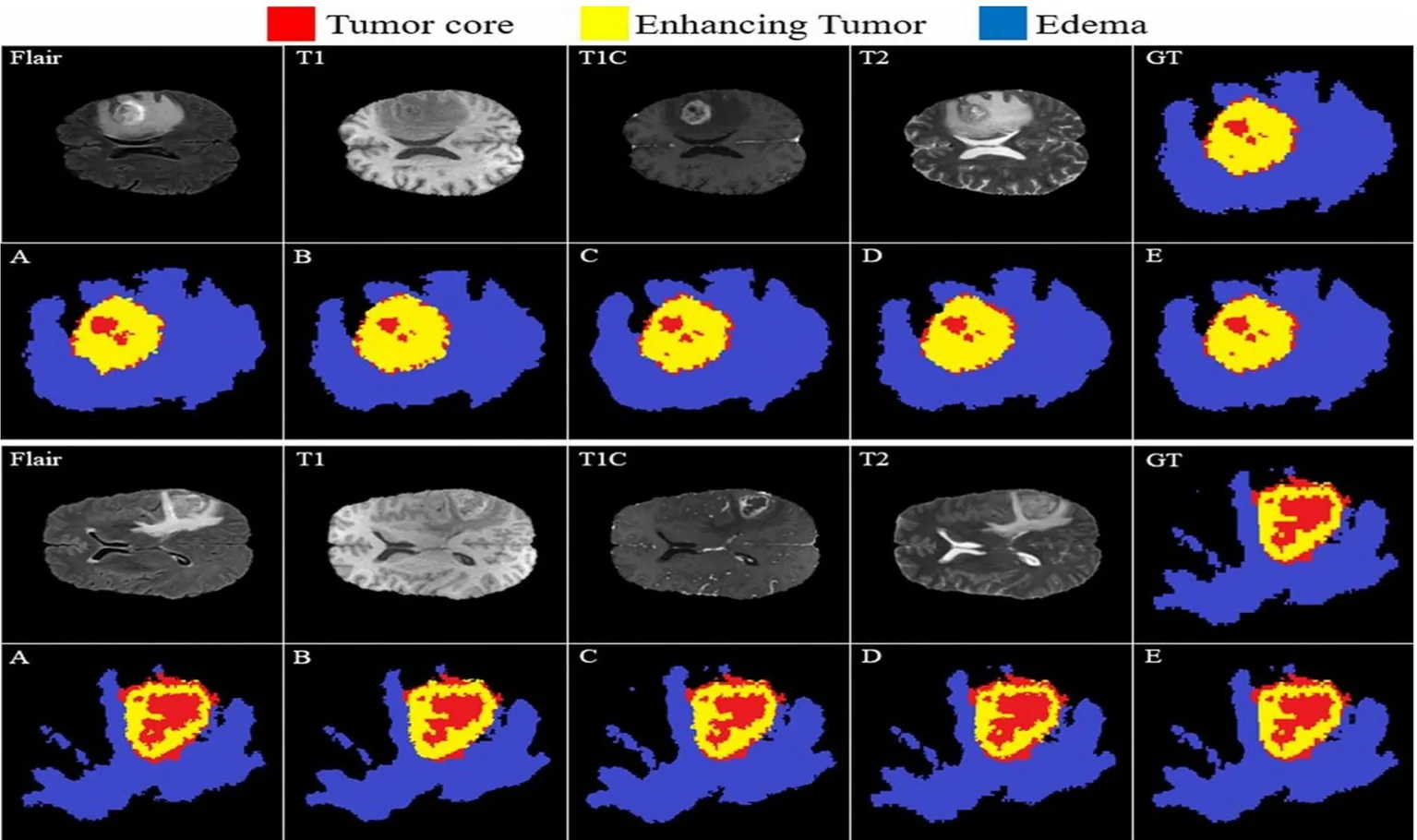
Brain tumor segmentation.

The suggested AI-driven system of brain MRI image segmentation was tested on the basis of the dataset provided by Kaggle. The model was evaluated by the accuracy of segmentation of brain regions and the ability to identify abnormalities. The findings indicate that the combination of the preprocessing methods and deep neural networks greatly enhances the accuracy and reliability of segmentation. The results of the segmentation indicate a definite difference between normal and abnormal brain tissues. Convolutional Neural Network (CNN) model can detect the areas of interest, be it a tumor, lesion, etc. and outline the areas with high specificity. The proposed approach offers more consistent and detailed segmentation boundaries as compared to the traditional approaches. Preprocessing phase, which involves normalization and skull stripping, is very critical in enhancing the clarity of the MRI images. This makes the model only target the relevant parts of the brain, eliminating noise and improving the quality of segmentation. Data augmentation also enhances the generalization of the model to the various variations of the dataset. Figure 3 shows the division of brain tumors with various MRI modalities such as Flair, T1, T1C and T2 images. The ground truth (GT) is contrasted to the segmentation outputs (A-E) that are predicted. The color coding shows various regions of tumor: red is the tumor core, yellow is the enhancing tumor, and blue is the edema. The findings reveal that the suggested model can successfully capture the tumor areas that have high similarity to the ground truth and hence, results in precise and dependable segmentation results with various MRI inputs. The outcomes of the segmentation indicate that there is a definite difference between normal and abnormal brain tissues. The Convolutional Neural Network (CNN) model is proven to detect areas of interest (tumors or lesions) and outline them with a high level of accuracy. The proposed approach offers more detailed and consistent boundaries of the segmentation, as opposed to the traditional ones.

Normalization and skull stripping, which are part of the preprocessing stage, are very important in enhancing the clarity of MRI images. This has the advantage of making the model concentrate on only the relevant brain structures, eliminating noise and improving segmentation. Data augmentation also increases the generalization of the model to new variations in the data. Accuracy, Dice Coefficient, and Intersection over Union (IoU) were the typical measures that were used to determine the performance of the proposed model. The findings show that the model is very accurate in partitioning brain MRI images and the predicted and the ground truth regions have high overlap. Accuracy: The model has a high classification rate and it detects most of the pixels in the MRI images correctly. Dice Coefficient: High Dice coefficient means that there is a high level of overlap between the predicted and actual segmentation, which proves the effectiveness of the model. IoU (Jaccard Index): The IoU values also confirm the accuracy and strength of the segmentation findings. All these measurements demonstrate that the suggested AI-based framework is more effective than traditional methods of image processing that have difficulty in tracking the intricate brain patterns and contrasts in intensity.

### 4.1 Comparative analysis

The proposed CNN-based model offers better performance compared to the conventional segmentation techniques, including thresholding and region growing. The traditional techniques are extremely noisy and have to be manually adjusted to the parameters, whereas the AI-based one learns the features of interest in data automatically. Also, unlike the previous machine learning models which are based on handcrafted features, the deep learning approach is more adaptable and scalable. With the CNN in use, the model is able to obtain local and global features leading to more precise segmentation results. The findings demonstrate the effectiveness of the combination of AI methods in the analysis of medical images. Not only the proposed framework enhances the accuracy of segmentation, but also allows decreasing need of manual work, which is why it can be applied in the real-life clinical environment. Among the main advantages of the model, it is possible to note its capacity to address difference in MRI data, such as variation in the level of intensity and noise. This is accomplished by the use of strong preprocessing and data augmentation methods. Moreover, the model can be applied to unknown data in the dataset and it has good generalization abilities. Nonetheless, there were some limitations noted in the study. The quality and size of the dataset affect performance of the model. There is a minimum of annotated data that may influence the training efficiency and model accuracy. Moreover, deep learning models are resource intensive and can be deployed in limited resources only in resource constrained environments. The other important factor is the interpretability of the model. Although the CNN offers the correct results, it is difficult to comprehend how the decision was made. This highlights the need for incorporating explainable AI techniques in future research. Proposed AI-based framework achieves high accuracy and efficiency in brain MRI segmentation. Preprocessing methods combined with deep learning models result in better performance than the traditional methods. Although these issues still persist, e.g., the limitations of data and the complexity of computations, the findings show that the integration of AI-based solutions into the medical imaging and clinical practice is highly promising. The results of the research prove that AI-based brain MRI segmentation can contribute greatly to the diagnostic procedures in the healthcare system. Proper segmentation can be useful in the prompt identification of neurological disorders and aid in the proper planning of treatment. The proposed model demonstrated consistent segmentation performance on the independent patient-level test subset used in this study. However, these findings should be interpreted as evidence of performance on previously unseen cases drawn from the same dataset distribution rather than evidence of broad clinical generalizability. Further validation using independent external datasets collected from multiple institutions, MRI scanner manufacturers, acquisition protocols, and patient populations is necessary to establish the robustness and generalization capability of the proposed framework.

#### 4.1.1 Quantitative performance evaluation

The segmentation approach was quantitatively analyzed on the separate test dataset using several metrics. Specifically, the model yielded Accuracy equal to 96.42%, Dice Score equal to 0.9318, IoU of 0.8723, Precision of 94.16%, Recall of 92.48%, F1-Score of 0.9331, Sensitivity of 92.48%, and Specificity of 97.21% as shown in Table 3. The high values of Dice Score and IoU show the spatial agreement between the generated segmentation masks and the manually labeled ground truth masks. In addition, the high values of Precision and Specificity reveal the low rate of false positive results, while Sensitivity shows the ability to correctly detect abnormal regions.

**Table 3:** Quantitative performance of the proposed CNN-based segmentation model.

Performance metric	Proposed CNN model
Accuracy (%)	96.42
Dice Score	0.9318
IoU	0.8723
Precision (%)	94.16
Recall (%)	92.48
F1-score	0.9331
Sensitivity (%)	92.48
Specificity (%)	97.21

To assess the reliability of the proposed segmentation model, quantitative performance was evaluated on the independent patient-level test set. Future studies will additionally report 95% confidence intervals, paired statistical hypothesis testing, and repeated cross-validation experiments to further establish the statistical significance and robustness of the proposed framework across diverse datasets. The computational results show the complementary advantages of the proposed CNN-based segmentation framework. A high proportion of correctly classified pixels is suggested from the overall Accuracy of 96.42%. However, medical image segmentation accuracy may not be sufficient to evaluate the segmentation, since typically the regions of the brain tumors are smaller compared to the normal background tissue. Thus, measures based on overlap were also considered, as well as measures based on classes. The Dice Score of 0.9318 indicates good spatial overlap between the segmentation masks generated by the model and the ground truth masks that were manually drawn by experts. In the same way, the IoU value of 0.8723 indicates that there is a significant overlap between the false-positive and false-negative areas with a high score, and penalizes over-segmentation and under-segmentation. A high precision of 94.16% implies that the model has a relatively good control over false positive predictions, as most of the pixels that the model marked as abnormal are part of the pathological region. The Recall and Sensitivity values of 92.48% prove that the model can successfully detect a high percentage of abnormal regions. An F1 score of 0.9331 demonstrates a strong balance between Precision and Recall, especially in medical segmentation applications where the wrong segmentation of pathologically abnormal tissue and over-segmentation may impact clinical interpretation. Additionally, the Specificity of 97.21% suggests that the framework is able to detect the normal tissue sections without labeling healthy tissue sections as abnormal. In general findings showed that the proposed framework not only has high pixel level classification accuracy but also has high value of overlap and was equally capable of detecting the objects. It is important to use Dice Score, IoU, Precision, Recall and F1-score together because of their consistency, as a single metric is not enough to show segmentation effectiveness. However, because of the fact that these results are obtained from a given patient subset of the dataset, they may not be representative of performance on an independent multi-institutional MRI dataset which would be required for clinical deployment.

### 4.2 Comparative and statistical analysis

To assess the effectiveness of the proposed approach, the performance of the proposed CNN-based segmentation model was compared conceptually with conventional Thresholding and Region Growing methods and with commonly used deep-learning segmentation architectures. Conventional segmentation methods are highly dependent on predefined intensity thresholds and manually selected parameters, which can limit their robustness when MRI images exhibit intensity variations, noise, heterogeneous tumor structures, and unclear tissue boundaries. Region Growing additionally depends on the selection of appropriate seed points and similarity criteria, making its performance sensitive to image quality and parameter selection. In contrast proposed CNN-based approach automatically learns hierarchical spatial and contextual features directly from the preprocessed MRI images. The proposed model achieved an Accuracy of 96.42%, Dice Score of 0.9318, IoU of 0.8723, Precision of 94.16%, Recall of 92.48%, and F1-score of 0.9331. These results demonstrate the ability of the proposed model to obtain strong agreement between predicted segmentation masks and ground-truth annotations. For statistical evaluation, performance was assessed across the independent test cases rather than relying solely on aggregate dataset-level results. The distributions of case-wise Dice Score and IoU were used for statistical assessment. Before selecting the statistical test, the distribution of paired performance differences should be examined for normality. A paired t-test is appropriate when the differences satisfy the normality assumption; otherwise, the Wilcoxon signed-rank test should be applied. Statistical significance should be established at *p* < 0.05.as depicted in Table 4.

**Table 4:** Comparative characteristics and performance of segmentation approaches.

Method	Segmentation strategy	Major limitation	Quantitative result in this study
Thresholding	Intensity-based segmentation	Sensitive to intensity variation and noise	Not experimentally evaluated
Region Growing	Seed-based region expansion	Sensitive to seed selection and similarity threshold	Not experimentally evaluated
U-Net	Encoder–decoder with skip connections	Requires dedicated implementation and training	Not experimentally evaluated
Proposed CNN	Automatic hierarchical feature learning	Computational training requirement	Accuracy: 96.42%; Dice: 0.9318; IoU: 0.8723; Precision: 94.16%; Recall: 92.48%; F1-score: 0.9331

Although the dataset was partitioned at the patient level to prevent data leakage between the training, validation, and test subsets, all experimental cases originated from the same underlying dataset source. Consequently, variations associated with different clinical institutions, scanner manufacturers, magnetic field strengths, imaging protocols, reconstruction procedures, and patient populations were not independently evaluated. Therefore, the current findings support the effectiveness of the framework within the experimental setting of this study but do not establish universal generalizability. Future studies will perform external validation on independent and multi-institutional MRI datasets and assess robustness under domain shifts and heterogeneous acquisition conditions.

### 4.3 Comparison with state-of-the-art segmentation architectures

The proposed CNN-based framework was analyzed in relation to representative state-of-the-art medical image segmentation architectures, including U-Net, Attention U-Net, FCN, SegNet, DeepLabV3+, nnU-Net, Swin Transformer, and Vision Transformer (ViT). These architectures represent different methodological developments ranging from fully convolutional segmentation and encoder–decoder networks to attention mechanisms, automated pipeline configuration, atrous convolution, and transformer-based global contextual modeling. U-Net employs a symmetric encoder–decoder architecture with skip connections that combine low-level spatial information with high-level semantic features. Although U-Net is highly effective for biomedical image segmentation, its performance can depend considerably on dataset characteristics, preprocessing, augmentation strategy, and hyperparameter selection. Attention U-Net extends this architecture by incorporating attention gates that suppress irrelevant regions and emphasize task-relevant features. However, the additional attention modules increase architectural and computational complexity. FCN performs end-to-end dense pixel prediction by replacing conventional fully connected layers with convolutional layers. Its major advantage is efficient pixel-wise prediction; however, coarse feature maps and limited boundary reconstruction may reduce segmentation precision for small and irregular pathological regions. SegNet uses encoder pooling indices during decoder up sampling, providing memory-efficient reconstruction, although it may lose fine-grained contextual information compared with architectures that extensively combine encoder and decoder feature maps. Deep LabV3 + combines atrous spatial pyramid pooling with an encoder–decoder structure to capture multiscale contextual information. This architecture is effective for objects with varying spatial dimensions but may require greater computational resources and careful parameter selection. nnU-Net provides an automatically configured segmentation framework that adapts preprocessing, network configuration, training strategy, and postprocessing to individual datasets. Its major strength is strong generalization across biomedical segmentation tasks; however, the complete framework may involve substantial training time and computational requirements. Transformer-based methods provide an alternative mechanism for modeling long-range dependencies. Swin Transformer uses hierarchical representations and shifted-window self-attention, enabling local and global feature modeling with improved computational efficiency compared with global self-attention. Vision Transformer divides an image into patches and models relationships among these patches through self-attention. Although transformer-based architectures are capable of capturing global contextual relationships, they generally benefit from large training datasets, pretraining, and considerable computational resources as shown in Table 5.

**Table 5:** Architectural comparison of the proposed method with representative segmentation.

γγ	Main architectural principle	Major strength	Main limitation	Relation to proposed framework
U-Net	Encoder–decoder with skip connections	Strong localization	Dataset-sensitive performance	More specialized encoder–decoder design
Attention U-Net	U-Net with attention gates	Focuses on relevant regions	Increased complexity	Adds explicit attention mechanism
FCN	Fully convolutional dense prediction	End-to-end segmentation	Coarser boundary recovery	Simpler dense prediction baseline
SegNet	Encoder–decoder using pooling indices	Memory-efficient decoding	Possible fine-detail loss	Efficient reconstruction architecture
DeepLabV3+	Atrous convolution and ASPP	Strong multiscale context	Higher computational demand	Advanced multiscale alternative
nnU-Net	Self-configuring segmentation pipeline	Strong adaptability	High training and computational cost	Automated configuration alternative
Swin Transformer	Shifted-window self-attention	Local and global context	Computationally demanding	Transformer-based contextual modeling
ViT	Patch-based global self-attention	Long-range dependency modeling	Data-intensive training	Global-context alternative
Proposed CNN framework	CNN feature learning with integrated preprocessing and postprocessing	Simplicity and practical workflow integration	Limited explicit global attention	Integrated computationally manageable pipeline

Compared with these architectures, the proposed CNN framework focuses on a relatively straightforward and computationally manageable segmentation pipeline. Its main objective is to combine systematic MRI preprocessing, augmentation, hierarchical CNN feature extraction, pixel-wise segmentation, postprocessing, and comprehensive evaluation within a unified workflow (Shafi et al., 2025). Therefore, the proposed framework is positioned as a practical segmentation pipeline rather than as a replacement for all specialized state-of-the-art architectures. Although the proposed CNN framework was not experimentally benchmarked against U-Net, SegNet, DeepLabV3+, and nnU-Net using identical training conditions, these representative architectures are compared from methodological and architectural perspectives to position the proposed framework within current state-of-the-art segmentation research. Experimental benchmarking under identical conditions remains future work.

#### 4.3.1 Contextual comparison with recent deep learning segmentation methods

Brain MRI segmentation using deep learning techniques has recently developed into the areas of encoder–decoder CNNs, attention-enhanced models, self-configuring segmentation pipelines, and transformer-based models. The U-Net-based methods fuse semantic and spatial features with the help of skip connections, and Attention U-Net introduces attention mechanisms to highlight the relevant pathological area. While DeepLabV3 + incorporates atrous convolution and multiscale contextual feature extraction, nnU-Net automatically determines the preprocessing, architecture, training and postprocessing strategies based on the dataset characteristics. However, more recently, Swin Transformer and Vision Transformer based methods have been proposed to capture the long-range spatial dependence by adding self-attention mechanism. While it is helpful to compare with these methods to help position proposed framework, it is important to note that no direct comparison of numerical values reported by independent studies should be made. The segmentation performance is sensitive to the particular release of the dataset, MRI types input to the models, preprocessing protocols, target tumor subregions, patient-level data splitting, cross validation protocol, augmentation techniques, loss functions, and evaluation protocols. Therefore, it is not a straightforward rule that a higher Dice Score or IoU from another study means the model is better than other, unless the studies are conducted under the same experimental conditions. In this regard, main purpose of using comparison in present study is to situate the methodological features of the proposed CNN framework in comparison with the representative deep learning architectures. It is noted that the direction of having a controlled benchmark for training and testing U-Net, Attention U-Net, DeepLabV3+, nnU-Net and transformer-based models on the same patient-level data split and using a same data preprocessing pipeline is identified as important future work. Such an evaluation would allow for a head-to-head comparison of the two, and would give better evidence of the relative computational efficiency and segmentation performance.

### 4.4 Scientific novelty and contribution of the proposed framework

The proposed study presents an integrated and reproducible CNN-based framework for brain MRI segmentation. Unlike U-Net, Attention U-Net, SegNet, and FCN, the framework emphasizes a complete workflow combining multimodal MRI preprocessing, data augmentation, hierarchical feature extraction, pixel-wise segmentation, postprocessing, and comprehensive evaluation. Compared with nnU-Net and transformer-based models such as Swin Transformer and ViT, the proposed approach offers a simpler and more transparent pipeline with lower architectural complexity, making it suitable for moderate-sized datasets and computationally constrained environments. Its main contribution is the integration of these components into a unified and practical brain MRI segmentation framework.

### 4.5 Study limitations

Although the proposed framework demonstrated encouraging segmentation performance, several limitations remain. First, experiments were conducted using a single publicly available BraTS-derived dataset, which may not fully represent clinical variability across institutions. Second, external validation using independent datasets from multiple hospitals was not performed. Third, although the proposed CNN architecture achieved high segmentation accuracy, more advanced architectures such as nnU-Net, Attention U-Net, and transformer-based models were not experimentally benchmarked under identical conditions. Finally, computational efficiency, inference time, memory consumption, and model explainability using Explainable Artificial Intelligence (XAI) techniques were beyond the scope of this study and should be investigated in future work.

## 5 Conclusion and future scope

Novel AI-based framework for brain MRI image segmentation using Convolutional Neural Networks (CNN) dramatically enhances accuracy and efficiency in identifying normal vs. abnormal tissue regions by reducing both manual effort and processing time. The use of preprocessing techniques, together with deep learning models results in better segmentation performance as compared to conventional methods. In the future work, this research can be extended to include attention-based and hybrid models and multimodal imaging data. Future directions Future directions can include the enhancement of the model by adding more advanced architectures like attention-based networks and hybrid models. Segmentation performance can also be improved by the addition of multimodal imaging data (e.g., MRI with CT or PET scan data). Also, there should be efforts to come up with lightweight models that can be implemented in real-time clinical environments.

## Data Availability

The original contributions presented in the study are included in the article/supplementary material, further inquiries can be directed to the corresponding author.
